# Antioxidant-Prooxidant Properties of a New Organoselenium Compound Library

**DOI:** 10.3390/molecules15107292

**Published:** 2010-10-21

**Authors:** Daniel Plano, Ylenia Baquedano, Elena Ibáñez, Iosu Jiménez, Juan Antonio Palop, Julian E. Spallholz, Carmen Sanmartín

**Affiliations:** 1 Department of Organic and Pharmaceutical Chemistry, University of Navarra, Irunlarrea, 1,E-31008 Pamplona, Spain; 2 Texas Tech University, Department of Nutrition, Lubbock, TX 79430, USA

**Keywords:** selenium, superoxide, diselenodipropionic acid, cytotoxicity

## Abstract

The present study describes the biological evaluation of a library of 59 organo-selenium compounds as superoxide (O_2_^─^) generators and cytotoxic agents in human prostate cancer cells (PC-3) and in breast adenocarcinoma (MCF-7). In order to corroborate that the biological activity for selenium compounds depends on the chemical form, a broad structural variety is presented. These structures include selenocyanates, diselenides, selenoalkyl functional moieties and eight newly synthesized symmetrically substituted dithioselenites and selenylureas. Eleven of the derivatives tested showed high levels of superoxide generation *in vitro* via oxidation of reduced glutathione (GSH) and nine of them were more catalytic than the reference compound, diselenodipropionic acid. Eighteen of the library compounds inhibited cell growth more than or similar to reference chemotherapeutic drugs in PC-3 and eleven were more potent cytotoxic agents than etoposide in the MCF-7 cell line. Considering both parameters (superoxide generation and cell cytotoxicity) compounds **B1, C6** and **C9** displayed the best therapeutic profiles. Considering that many diselenide compounds can generate superoxide (O_2_^─^) *in vitro* via oxidation of GSH and other thiols, the analogue **B1**, that contains a diselenide moiety, was selected for a preliminary mechanistic investigation, which . revealed that **B1** has apoptogenic effects similar to camptothecin mediated by reactive oxygen species (ROS) in lymphocytic leukemia cells (CCRF-CEM) and affected the MCF-7 cell-cycle in G_2_/M and S-phases.

## 1. Introduction

**Figure 1:**
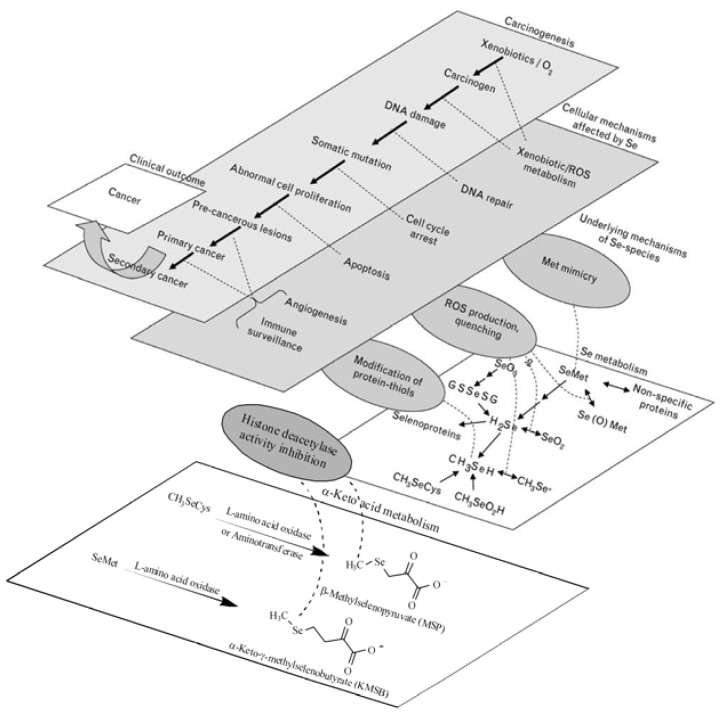
A multi-tiered model of Se anticarcinogenesis.

Selenium (Se) is an important nutritional trace element involved in different physiological functions with antioxidative, antitumoral and chemopreventive properties [1]. There is increasing evidence that dietary Se intakes are sub-optimal in the populations of many countries and that human cancer mortalities would significantly decline with supplementation [2], although various authors [3,4] have considered some potential limitations to this fact. The essential trace element Se is the catalytic cofactor of important endogenous antioxidative systems of the human body, attracting more and more attention of laypersons and research groups [5]. Se is present in 25 human selenoproteins, many of them involved in anti-oxidant defense systems and in cancer prevention [6]. Selenoproteins include enzymes, such as the glutathione peroxidases (GPx), thioredoxin reductases (TrxR) and iodothyronine deiodinases (ID) [7]. It is well established [8] that these Se enzymes play a key role in redox regulation as modulators of reactive oxygen species (ROS). Reactive oxygen species are constantly generated and eliminated in all biological systems, and they play important roles in a variety of normal biochemical functions and abnormal pathological processes. These species, including superoxide (O_2_^─^), hydrogen peroxide (H_2_O_2_) and hydroxyl radicals (OH·) are known to mediate apoptosis acting as inducers of cancer chemoprevention and as therapeutic agents derived from catalytic selenium compounds. Excess intracellular ROS may attack cellular membrane lipids, proteins, and DNA, inhibiting their normal functions by causing oxidative damage. This is one of the proposed anticancer mechanisms of selenium compounds [9]. In spite of these facts, the effectiveness of the incorporation of Se into anticarcinogenic compounds can be conceptualized as a multi-tiered process whereby chemical/biochemical actions of its metabolites, such as methylselenol (CH_3_SeH/CH_3_Se^-^), hydrogen selenide (H_2_Se, HSe^−^) or other selenide anions (RSe^−^) arise (Figure 1) [9,10,11,12]. 

**Figure 2:**
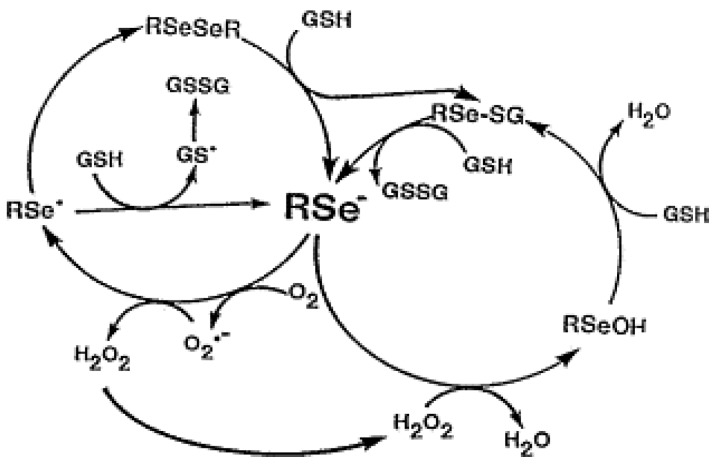
Redox cycling of methylselenide and other selenides (RSe^−^).

These species can each undergo oxidation/reduction, according to Figure 1 and Figure 2. For example GSSe^−^ and HSe^-^ may affect the redox cycle, helping to deplete GSH and produce ROS as superoxide, hydrogen peroxide and the hydroxyl radical. Other selenium compounds have been reported to generate superoxide and H_2_O_2_ *via* oxidation of GSH following diselenide reduction and methyl selenide (CH_3_Se^−^) formation (Figure 2). Numerous research studies have shown that these redox effects depend upon the dose and the chemical form of the selenium compound [13,14,15]. 

**Figure 3:**
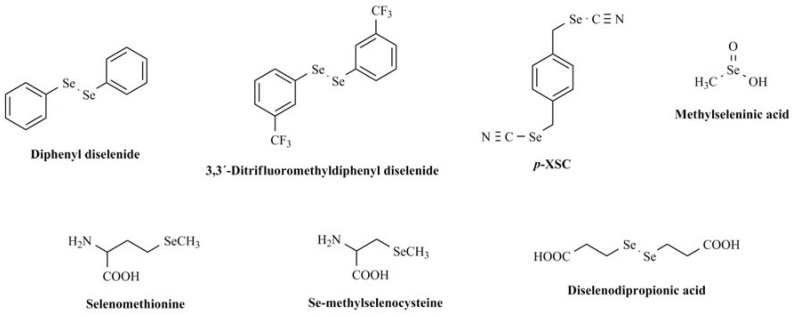
Chemical structures of some representative selenium compounds with antioxidant properties.

In the last few years, a number of novel synthetic organoselenium compounds have been synthesized for their use as antioxidants in medicinal chemistry. In addition, many of these derivatives have exhibited antitumoral activities. Evidence indicates several different mechanisms for this Se activity. The properties of diselenides have received significant attention, particularly with the discovery that these compounds possess a mode of antioxidant action similar to that of glutathione peroxidase. Such antioxidant diselenides include diphenyl diselenide [16,17], 3,3´-diselenodipropionic acid [18,19], binaphthyl diselenide [20] and 3,3´-ditrifluoromethyldiphenyl diselenide [21]. Other chemical forms of Se that are also able to protect against oxidative damage are selenomethionine, methylseleninic acid, dimethyldiselenide [22] and Se-methylselenocysteine. These methylated selenium derivatives [23] can undergo the metabolic generation of the monomethylated selenium species, methylselenide, which can undergo oxidation/reduction processes. Selenocyanate derivatives also represent an important class of agents with antioxidative properties; as 1,4-phenylenebis-(methylene)selenocyanate (*p*-XSC) [24] or monoselenocyanate [25]. Redox activity can be correlated with inhibition of tumor formation. Drake [26], contrary to the prevailing thought, presented evidence that Se in the +4 oxidation state is more effective as an anticarcinogenic agent than Se in the +2 oxidation state. Examples of biologically active antioxidant selenium compounds are illustrated in Figure 3. 

Much experimental evidence suggests that modification of organoselenium compounds can have a profound effect on their chemical and therefore on their biological activity. From this viewpoint, and taking into account that during the last several years, the principal interest in our research group has been the study, syntheses and biological evaluation of a variety of compounds containing Se as candidates for new antineoplastic agents [27,28,29,30,31], the objectives of the present study were twofold. Firstly, an evaluation was planned in order to choose compounds that present a dual activity: superoxide generation complemented with cytotoxicity. Several studies have found a good correlation between the redox potential of selenium compounds and their cytotoxicity [9,10,13].

Secondly, the synthesis and biological evaluation of an original series consisting of a small group of compounds which possess Se in the +4 states oxidation with the aim of establishing that these Se +4 derivatives can catalyze the oxidation of thiol groups generating superoxide [26]. Therefore the biological evaluation as potential antioxidant and cytotoxic agents of a library of 59 selenium compounds is presented, eight of them newly synthesized selenylurea and dithioselenite derivatives.

## 2. Results and Discussion

### 2.1. Chemistry

**Figure 4:**
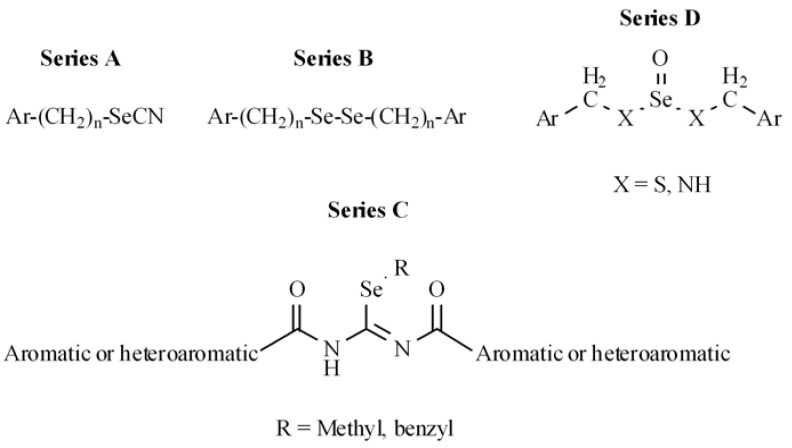
General chemical structures for the series of compounds studied **A**-**D**.

The compounds reported in this paper correspond to four different chemical series (**A**-**D**) as illustrated in Figure 4. 

The preparation of the series **A** compounds began with the reaction and addition of selenium dioxide to malononitrile in dimethyl sulfoxide (DMSO) at room temperature, followed by reaction with the corresponding aminoaryl derivatives or by the reaction of haloarenes with potassium selenocyanate in acetone under reflux conditions, according to a literature procedure [32]. Conversion of the derivatives in series **A** to the corresponding series **B** compounds was carried out by reduction with sodium borohydride in ethanol, according to the synthetic method previously described [32].

The imidoselenocarbamate derivatives in series **C** were synthesized following previously published methods [27,33], starting from the appropriate Se-alkyl imidoselenocarbamate hydroiodide and the corresponding acyl chloride.

**Scheme 1:**
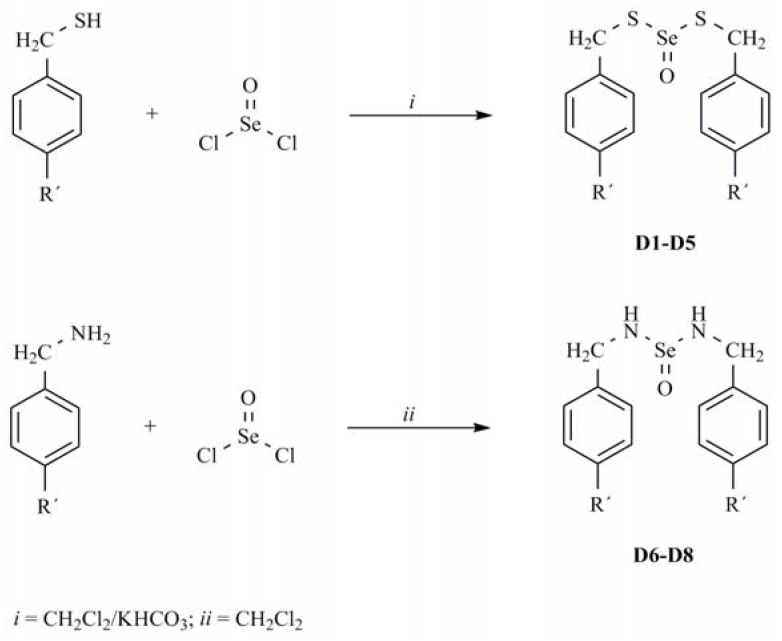
Synthetic routes for compounds related to dithioselenites and selenylureas (series **D**).

To explore the effect of substitution of Se +2 with Se in the +4 oxidation new compounds related to dithioselenites and selenylureas (compounds within series **D**) were synthesized. The synthetic routes for the preparation of these series **D** compounds are presented in Scheme 1.

Dithioselenites **D1-D5** were prepared from the corresponding functionalized benzylmercaptan and selenium oxychloride (SeOCl_2_) in dichloromethane in the presence of potassium bicarbonate [34]. When the mercaptans were not commercially available they were obtained from the corresponding benzyl halide and sodium hydrosulfide by refluxing in ethanol [35]. For the preparation of selenylureas **D6-D8** the key benzylamines were subjected to reaction with SeOCl_2_ under a nitrogen atmosphere. This methodology was tentatively performed in order to obtain derivatives with strong electron withdrawing substituents. Despite numerous efforts, all attempts at introducing these strong electron withdrawing moieties using various reaction conditions failed. A possible explanation to this fact could be that the amine function in the presence of SeOCl_2_ generates a chloroamine group that can progress to an aldehyde group through an imine intermediate which interferes with the main desired addition reaction. This effect is even more marked when the substituent in the aryl ring are strongly electron withdrawing. For this reason the yield for the derivative **D8** in the **D** series was very poor (8%) and for other attempted benzylamines (NO_2_, CN) the yields were negligible and the products could not be isolated. The physical constants of compounds **D1**-**D8** are listed in Table 1. The IR, NMR and MS spectral data obtained for the newly synthesized compounds all correspond to the theoretical chemical structures. 

### 2.2. Biological Evaluation

All of the selenium compounds described in this paper (**A1**-**A13**, **B1**-B13, **C1**-**C25** and **D1**-**D8**) were screened for their ability to generate superoxide by the oxidation of GSH by a lucigenin dependent chemiluminescent (CL) assay [36]. Their *ex vivo* cytotoxic activity against the human prostate cancer (PC-3) and breast adenocarcinoma (MCF-7) cell lines using the MTT (3-[4,5-dimethylthiazol-2-yl]-2,5-diphenyltetrazolium bromide) protocol was followed [37]. 

#### 2.2.1. Superoxide anion scavenging activity

The superoxide anion is considered to be biologically important since it can dismutate to form stronger oxidative species, H_2_O_2_, singlet oxygen and hydroxyl radicals. In the PMS/NADH-NBT (phenazine methosulphate/nicotinamide adenine dinucleotide-nitroblue tetrazolium) system, superoxide anions are derived from the dissolved oxygen by a PMS/NADH coupling reaction, which can specifically reduce NBT. The decrease of NBT absorbance at 560 nm indicates the consumption of superoxide anions in the reaction mixture [36]. Experiments were carried out using a concentration of 4 µg/µL for all the compounds and the results are shown in Table 2 (compounds of series A and B), Table 3 (compounds of series C) and Table 4 (compounds of series D). Diselenodipropionic acid, known to affect the redox cycle generating superoxide, was used as the reference compound.

#### 2.2.2. Cytotoxic activity in PC-3 and MCF-7

Initially, the compounds were evaluated for their *ex vivo* cytotoxic activity against human prostate cancer (PC-3, ATCC, Manassas, VA) and breast adenocarcinoma (MCF-7, ATCC, Manassas, VA) cell lines. These cell lines were selected as there are many clinical trials in the literature showing the activity of selenium compounds in the reduction of hormone dependent cancer [38,39]. Cytotoxicity results were tabulated as IC_50_ values. All experiments were independently performed at least three times and the values calculated after 72 hours of selenium compound exposure (concentrations of 2, 5, 7 and 10 μM were employed throughout). The results are shown in Table 2 (compounds of series A and B), Table 3 (compounds of series C) and Table 4 (compounds of series D). Methylseleninic acid (MSA) in PC-3 and etoposide in PC-3 and MCF-7 were used as controls in the cytotoxic assays [40,41,42].

All compounds of the selenium library that presented chemiluminescence units (CLU) values more than tenfold the control have been considered as potent superoxide generators. The results presented in Table 2 indicate that three compounds (**B1**, **B6**, and **A10** in this decreasing CLU order) were potent superoxide generators. Comparison of the results with the values for the standard diselenodipropionic acid showed that compound **B1** was 1.5 times more active. It was observed that six compounds (**A1**, **A2**; **A7**, **A12**, **B1** and **B13**) possessed cytotoxic activity against PC-3 cells, of which four (**A2**, **A12**, **B1** and **B13**) were more active than methylseleninic acid and etoposide. Compound **B1** was the most potent (IC_50_ = 1.7 μM) and was five times more active than standard methylseleninic acid (IC_50_ = 8.4 μM) and eight times more active than etoposide (IC_50_ = 13.6 μM), an agent used in the treatment of prostate cancer. Moreover, two derivatives (**A12** and **B1**) were more potent cytotoxic agents than standard etoposide (IC_50_ = 17.5 μM) in MCF-7.

The results obtained for imidoselenocarbamate derivatives (Table 3) showed that three compounds, which presented a furyl (**C6** and **C16**) and thienyl (**C9**) heteroaromatic rings, were potent superoxide generators with CLU values higher than the control reference standard. Furthermore, 13 compounds (**C1-C10** and **C12-C14**) possessed cytotoxic activity and 11 (**C2**, **C4-C10**, **C12-C14**) of them presented IC_50_ values lower than MSA or etoposide in PC-3. In addition, considering MCF-7 cells eight of them (**C1-C5**, **C8-C10**) were more active than etoposide, highlighting **C3** and **C10** which showed IC_50_ values below 1 μM. It was also observed that selenomethyl derivatives (**C1-C15**) were more active as cytotoxic agents than selenobenzyl analogues (**C16-C25**). These results reinforced the earlier hypothesis concerning the determinant role of the selenomethyl group as a scaffold for the biological activity of these types of compounds. The likely explanation for this finding is that compounds with a selenomethyl moiety act as methylselenol precursors which generate superoxide by redox cycling. It has been suggested that methylselenol is the critical Se metabolite for anticancer activity of the dietary selenoamino acids. [43,44,45] so as the selenoketo acids [12] generated from metylselenocysteine and selenomethionine through amino acid oxidases and aminotransferases.

The compounds of series **D** were the most potent superoxide anion generators with five active compounds (**D2-D5** and **D8**), especially compound **D5**, with an average value of 4,743 CLU/30 seconds. Compounds therefore with Se in the +4 oxidation state, like selenite, seemed to be more active as superoxide generators than Se compounds in the +2 oxidation state. These results are according to those reported in the literature [26]. However, only one of these derivatives (**D6**) presented a significant cytotoxic effect in PC-3 and MCF-7 cell lines.

Taking together all the superoxide generating and cytotoxicity results, the evaluated selenium compounds presented four types of behaviour: a) compounds both inactive as superoxide generators and non-toxic xenobiotics to cancer cells, (**A3-A6**, **A8**, **A9**, **A11**, **A13**, **B2-B5**, **B7-B12**, **C15**, **C17-C25**, **D1** and **D7**) b) superoxide generating compounds but non-toxic xenobiotics to cancer cells, (**A10**, **B6**, **C16**, **D2-D5** and **D8**) c) compounds inactive as superoxide generators but cytotoxic xenobiotics to cancer cells (**A1**, **A2**, **A7**, **A12**, **B13**, **C1-C5**, **C7**, **C8**, **C10**, **C12-C14** and **D6**) and d) both superoxide generators and cytotoxic xenobiotics to cancer cells (**B1**, **C6**, **C9**). Taking into account that one of the objectives of the present study was the selection of organoselenium compounds with dual biological activity, the ability to induce superoxide generation and show cytotoxic activity, **B1**, **C6** and **C9** were selected for further study as the most interesting selenium derivatives. To gain further insight into the mechanisms of action of these active compounds, compound **B1** was also selected for an extensive study because it is a diselenide derivative and this moiety has been postulated as one of the important species in redox cycling (Figure 2). In addition, this compound, bis(4-amino-phenyl)diselenide exhibited cytotoxic activity in other cell lines (CCRF-CEM, IC_50_ = 9.0 μM; HT-29, IC_50_ = 9.8 μM). Various *in vivo* studies [18,20,21] have reported the diselenide moiety as a protective and antigenotoxic agent. Therefore, based on the chemical analogy between compound **B1** and the diselenides above mentioned, **B1** emerge as a potential candidate for *in vivo* studies.

#### 2.2.3. Apoptosis

**Figure 5:**
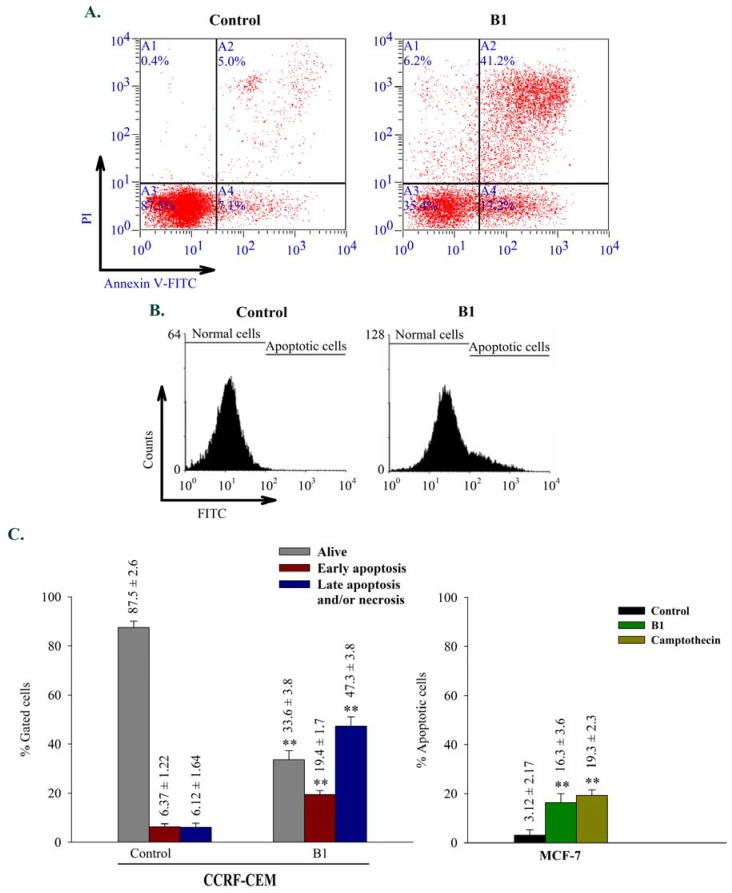
Apoptotic effects of compound **B1** and camptothecin at 10 or 25 μM on CCRF-CEM and MCF-7 cells for 24 and 48 h, respectively. (**A**) Apoptotic effects of compound **B1** on CCRF-CEM were assessed by Flow Cytometry analysis after staining with Annexin V-FITC. Annexin V staining is represented on the x-axis and PI staining is represented on the y-axis. The representative results of three independent experiments are shown; (**B**) Apoptotic effects of compound **B1** were assessed by Flow Cytometry analysis using the *Apo-Direct* kit, based on the TUNEL assay; (**C**) Percentage of apoptotic and or necrotic cells after compound **B1** or camptothecin treatment were calculated from the flow cytometry results. Each bar represents the mean of three independent experiments and the error bars indicate standard deviation. ** p < 0.01 with respect to the control.

Recent evidence indicates that ROS may function as intracellular messengers to modulate apoptosis and apoptotic responses have been demonstrated in many types of cancer cells treated with selenium compounds [9,11,46,47]. For this reason it was decided to assess whether this superoxide generating activity was related to their ability to induce apoptosis. **B1,** one of the most active compounds as a superoxide generator and cytotoxic agent, was further analyzed for apoptosis in MCF-7 cells. The apoptotic status of the cells after 48 hours of treatment with 25 μM of the compound **B1** was determined using the Apo-Direct kit (BD Pharmingen) based on the TUNEL assay as described by the manufacturer. Camptothecin was used as a positive control. The results obtained are shown in Figure 5. As can be seen, **B1** exerted apoptogenesis similar to camptothecin. The apoptogenic action of **B1** was confirmed to occur in lymphocytic leukemia cells (CCRF-CEM) (Figure 5). The apoptotic status of CCRF-CEM cells was confirmed by measuring the exposure of phosphatidylserine on the cell membranes using the Annexin V-FITC Kit (BD Pharmingen) [48]. After 24 hours of treatment with 10 μM of **B1** both apoptopic and necrotic effects were observed in the CCRF-CEM cells.

#### 2.2.4. Effects on cell cycle progression

Cell cycle arrest is one consequence of treatment with many anticancer drugs, including camptothecin. In an effort to ascertain whether compound **B1** could affect the cell cycle progression in MCF-7 cells, these cells were treated with 25 μM of **B1** for 48 h and cell cycle progression was determined by flow cytometry analysis. 

**Figure 6:**
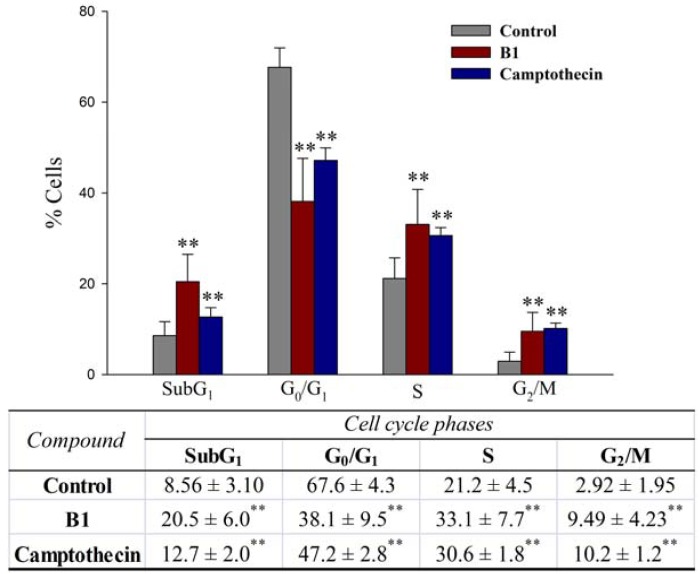
Cell cycle profile of MCF-7 cells after 48 h treatment with compound **B1** and camptothecin at 25 μM. Each bar represents the mean of three independent experiments and error bars indicate the standard deviation. ** p < 0.01 with respect to the control.

As shown in Figure 6, DNA flow cytometric analysis indicated that treatment of the cells with compound **B1** triggered a marked change in the MCF-7 cell cycle distribution. This compound induced cell accumulation in S and G_2_/M phases, accompanied by a diminution of cell population in the G_0_/G_1_ phase and a significant increase in SubG_1_ (which is representative of cells with fragmented DNA). It is noteworthy that cell cycle distribution analysis for compound **B1** is in total agreement with the apoptosis results obtained in MCF-7 cells. In summary, **B1** affects cell-cycle checkpoints in G_2_/M and S-phases causing a reduction of cell-cycle progression along with a cellular transition into apoptosis. 

#### 2.2.5. Effects on reactive oxygen species generation (ROS)

**Figure 7:**
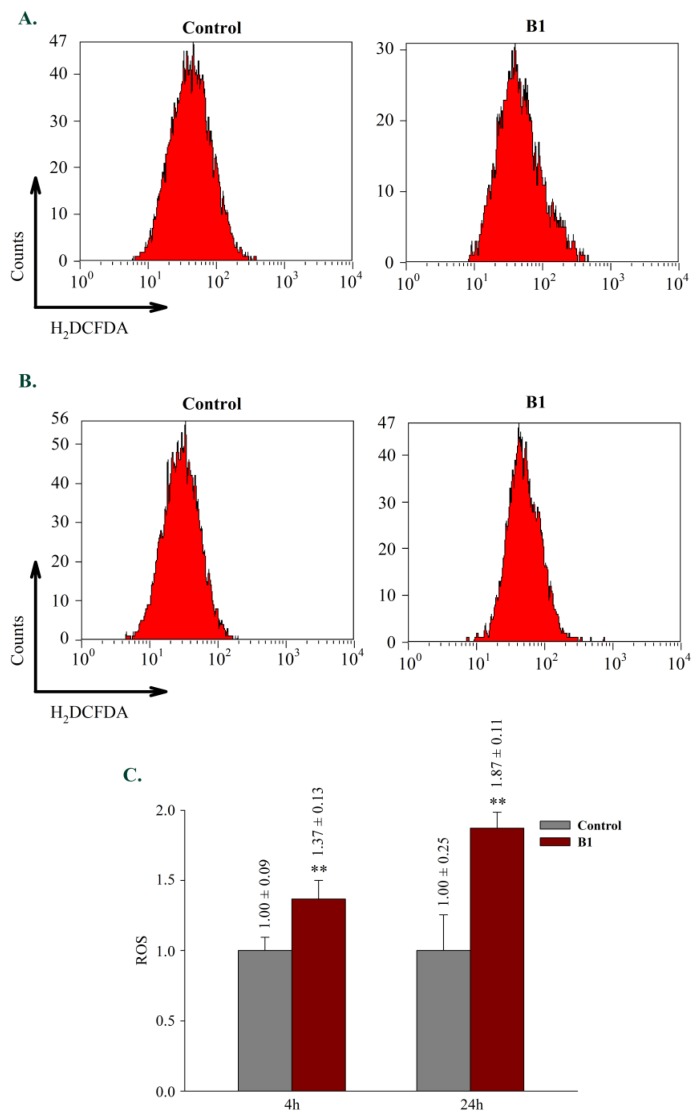
Intracellular content of ROS in CCRF-CEM cells after 4 and 24 h treatment with compound **B1** at 25 μM. (**A**) Flow Cytometry analysis is shown for 4 h treatment with compound **B1** or vehicle (control) for the representative results of three independent experiments; (**B**) Flow Cytometry analysis is shown for 24 h treatment with compound **B1** or vehicle (control) for the representative results of three independent experiments; (**C**) Each bar represents the mean of three independent experiments and the error bars indicate the standard deviation. ** p < 0.01 with respect to the control.

It is well established [8] that Se plays a key role in redox regulation as a modulator of reactive oxygen species (ROS) and numerous investigations have documented that oxidative stress-mediated cellular changes are frequently induced following exposure to cytotoxic drugs, UV or γ-radiation [49,50]. Therefore, we examined whether ROS generation might be involved in the induction of apoptosis by compound **B1** in the CCRF-CEM cell line. As shown in Figure 7, compound **B1** induced a significant increase in the intracellular content of ROS in CCRF-CEM cells after 4 and 24 h treatment at 25 μM. Moreover, the intracellular content of ROS seemed to be time-dependent, *i.e.* ROS increased with time. These results suggest the possible role of oxidative stress in cell death induced by compound **B1** in the CCRF-CEM cell line. Puntel *et al*. [51] have reported a mitochondrial dysfunction mediated by thiol oxidation induced by diphenyl diselenide. This could be a possible explanation for the oxidative stress induced by compound **B1**, but further studies need to be performed.

## 3. Experimental

### 3.1. General

Melting points for all the compounds of the selenium library were determined by differential scanning calorimetry using a Perkin-Elmer DSC Diamond and with a Mettler FP82 + FP80 thermomicroscopy (Greifense, Switzerland). The ^1^H-NMR spectra were recorded on a Bruker 400 UltrashieldTM (Rheinstetten, Germany), using TMS as the internal standard (Table 5). The IR spectra were recorded on a Thermo Nicolet FT-IR Nexus in KBr pellets (Table 5). Elemental microanalyses were carried out on vacuum-dried samples (Table 1) using an Elemental Analyzer (LECO, CHN-900 Elemental Analyzer). Silicagel 60 (0.040–0.063 mm) 1.09385.2500 (Merck KGaA, 64271 Darmstadt, Germany) was used for Column Chromatography and Alugram® SIL G/UV254 (Layer: 0.2 mm) (Macherey-Nagel GmbH & Co. KG. Postfach 101352. D-52313 Düren, Germany) was used for Thin Layer Chromatography. Chemicals were purchased from E. Merck (Darmstadt, Germany), Scharlau (F.E.R.O.S.A., Barcelona, Spain), Panreac Química S.A. (Montcada i Reixac, Barcelona, Spain), Sigma-Aldrich Química, S.A., (Alcobendas, Madrid, Spain), Acros Organics (Janssen Biological activity assays).

### 3.2. General Procedure for Preparation of 4-Mercaptomethylbenzonitrile and 4-Nitrophenylmethanethiol

To a solution of the corresponding 4-bromomethylbenzonitrile (20.13 mmol) or 4-nitrobenzyl bromide (20.13 mmol) and ethanol (50 mL) was added sodium hydrosulfide (44.66 mmol). The mixture was stirred at its refluxing temperature for 16 h and evaporated. The residue was triturated with water (100 mL) and extracted with dichloromethane (3 × 50 mL). Combined organic phases were washed with water (100 mL) and dried with anhydrous Na_2_SO_4_. Solvent was removed in vacuo and the resulting crude product was purified by washing with ethyl ether (4 × 25 mL). 

*4-mercaptomethylbenzonitrile:* Yield: 79%. IR (KBr): 2226 (s, CN); ^1^H-NMR (400 MHz, DMSO-*d*_6_, δ): 2.15 (s, 1H, SH); 3.85 (d, 2H, CH_2_); 7.43 (d, 2H, H_2_, H_6_, *J*_2-3_ = 8 Hz); 7.56 (d, 2H, H_3_, H_5_, *J*_3-2_ = 8 Hz). Anal. Calcd. for C_8_H_7_NS_2_ (%): C, 64.42; H, 4.70; N, 9.40. Found: C, 64.33; H, 4.75; N, 9.38.

*4-nitrophenylmethanethiol:* Yield: 64%. IR (KBr): 1.348 (w, C-NO_2_); ^1^H-NMR (400 MHz, DMSO-*d*_6_, δ): 2.14 (s, 1H, SH); 3.89 (d, 2H, CH_2_); 7.70 (d, 2H, H_3_, H_5_, *J*_3-2_ = 8.6 Hz); 8.17 (d, 2H, H_2_, H_6_, *J*_2-3_ = 8.6 Hz). Anal. Calcd. for C_7_H_7_NO_2_S_2_ (%): C, 49.70; H, 4.14; N, 8.28. Found: C, 49.39; H, 4.38; N, 7.95.

### 3.3. General Procedure for Preparation of Dithioselenites ***D1**-**D5***

To a solution of the corresponding mercaptan (13.50 mmol) in dry dichloromethane (12 mL) was added KHCO_3_ (0.6 g) and molecular sieves. The suspension was stirred for 1 h under nitrogen at 0°C and then was added SeOCl_2_ (0.5 mL). The mixture was maintained by stirring for 5 h at room temperature and water (50 mL) was added and extracted with dichloromethane (3 × 50 mL). Combined organic phases were washed with water (100 mL) and dried with anhydrous Na_2_SO_4_. Solvent was removed in vacuo and the resulting crude product was purified by recrystalization from methanol. According to this procedure the following compounds were synthesized: *N,N´*-bis-(*p*-chlorophenyl-methyl)-dithioselenite (**D1**), *N,N´*-bis-(p-methoxyphenylmethyl)-dithioselenite (**D2**), *N,N*´-bis-(*p*-trifluoromethylphenylmethyl)-dithioselenite (**D3**), *N,N’*-bis-(*p*-cyanophenylmethyl)-dithioselenite (**D4**) and *N,N’*-bis-(*p*-nitrophenylmethyl)-dithioselenite (**D5**).

### 3.4. General Procedure for Preparation of Selenylureas (**D6-D8**)

To a solution of the corresponding benzylamine (15 mmol) in dry dichloromethane (12 mL) under nitrogen atmosphere was added SeOCl_2_ (0.2 mL). The mixture was stirred for 3 h at room temperature until a black solid was formed. The residue was filtered. The organic layer was removed *in vacuo* and the crude material was washed with ethyl ether (3 × 15 mL) and re-crystallized from ethanol. According this procedure the following compounds have been synthesized: *N,N´*-bis-(*p*-chlorophenylmethyl)-selenylurea (**D6**), *N,N´*-bis-(*p*-methoxyphenylmethyl)-selenylurea (**D7**) and *N,N´*-bis-(*p*-trifluoromethylphenylmethyl)-selenylurea (**D8**).

### 3.5. Biological Activity Assays

#### 3.5.1. Superoxide anion generating activity 

The control chemiluminescent (CL) assay cocktail without substrates or GSH was made using a 0.05 M sodium phosphate buffer (pH 7.4) containing 20 μL lucigenin/mL from a stock solution of 1.0 mg/mL lucigenin in distilled water. The assay cocktail with thiol contained 1.0 mg GSH/mL. To aliquots of thiol (500 μL) containing assay cocktail were added each selenium compound in DMSO (20 μL). The concentration of each compound was no more than 4 μg/uL or otherwise saturated <4 μg/μL as not all compounds fully dissolved in the DMSO. The samples were added in one 20 μL portion from a 50 μL Eppendorf pipette directly to the chemiluminescent tube into a Los Alamos Diagnostics Model 535 luminometer containing an attached LKB 2209 multitemp recirculating water bath. Chemiluminescent data were recorded at 25 degrees C in 30-second integrated units over a period of up to 20 min. There was a3-second instrumental delay between integrations. Additional details of this assay including the quenching of chemiluminescence generated by superoxide dismutase have been previously reported [21]. This CL assay is quantitative (correlation coefficient, r = 0.99; P < 0.001) in generating CL for small amounts of redox cycling selenium compounds [21]. 

#### 3.5.2. Cytotoxic activity in PC-3 and MCF-7

PC-3 and MCF-7 cells were seeded in 96-well plates (Millipore, Eschborn, Germany) at a density of 1.5 × 10^3^ and 10^4^ cells per well, respectively. The cells were incubated at 37°C under 5% CO_2_ overnight prior to the addition of the compounds. All compounds were dissolved in DMSO at a concentration of 0.01M. Sterile filtration of the compounds was achieved using 0.2 μm filter disks. Serial dilutions with supplemented medium were prepared daily to a final concentration of 2, 5, 7 and 10 µM in cell culture. After 3 days of incubation, MTT solution (5 mg/mL in PBS, 10 μL) was added to the cells in each well and then incubated for an additional 4 h at 37°C. The absorbance of formazan at λ = 570 nm was measured on a Polarstar Galaxy plate reader (BMG LabTechnologies GmbH). The percentage of viable cells was calculated to obtain IC_50_-values. PC-3 and MCF-7 cells were cultured under standard conditions (Dulbecco’s RPMI 1640 medium, with GlutamaxTM 1, Invitrogen supplemented with 10% fetal bovine serum, Fetalclone III, SH30109.03, HYCLONE and 1% Penicillin-Streptomycin, Invitrogen). 

#### 3.5.3. Apoptosis and cell cycle

MCF-7 cells are human breast adenocarcinoma whose apoptotic status and cell cycle analysis of the cells were determined using the Apo-Direct kit (BD Pharmingen), based on the TUNEL assay under the conditions as described by the manufacturer. The apoptotic status of leukemia CCRF-CEM cells was evaluated by measuring the exposure of phosphatidylserine on the cell membranes using the Annexin V-FITC kit (BD Pharmingen). Briefly, 5 × 10^5^ cells were pelleted and washed in PBS. Cells were then stained with Annexin V-FITC and propidium iodide for 15 min at 4°C in the dark and analyzed using a Coulter Epics XL flow cytometer (Becton Dickinson). 

#### 3.5.4. Measurement of reactive oxygen species 

The intracellular accumulation of ROS was determined using the fluorescent probes 2′,7′-dichlorodihydrofluorescein diacetate (H_2_DCFDA). Briefly, CCRF-CEM cells were incubated with compound **B1** for 4 and 24 h. The cells were stained with 10 μM H_2_DCFDA for 60 min at 37°C and the fluorescence was measured at the desired time intervals by flow cytometry. The ROS generation was assessed by the dichlorofluorescein fluorescence intensity (FL-1, 530 nm) from 10,000 cells with a Coulter Epics XL flow cytometer (Becton Dickinson).

## 4. Conclusions

Eight new organoselenium derivatives **D1**-**D8** have been prepared and fifty-nine compounds related to a series of selenium derivatives have been evaluated for their biological activities as superoxide generators and cytotoxic agents. Three distinct cell lines (PC-3, MCF-7 and CCRF-CEM) were used in the biological evaluation of the derivatives. Thirteen of the selenium compounds (**A2**, **A9**, **A10**, **B1**, **B6**, **C6**, **C9**, **C16**, **D2**, **D3**, **D4**, **D5** and **D8**) were of interest as superoxide generators determined by a chemiluminescent assay. It is noteworthy that some of these were catalytically more active (**B1**, **C6**, **C9**, **C16**, **D2**, **D3**, **D4**, **D5** and **D8**) than the reference compound diselenodipropionic acid. Eighteen compounds (**A1**, **A2**, **A7**, **A12**, **B1**, **B13**, **C2**, **C4**, **C5**, **C6**, **C7**, **C8**, **C9**, **C10**, **C12**, **C13**, **C14** and **D6**) and eleven compounds (**A12**, **B1**, **C1**, **C2**, **C3**, **C4**, **C5**, **C8**, **C9**, **C10** and **D6**) inhibited cell growth in PC-3 and MCF-7 cell lines, respectively, presenting values for IC_50_ lower than or similar to the standard anticancer agents used (etoposide and/or methylseleninic acid). Considering the chemical and biological data as a whole, with the aim in mind of finding new selenium compounds that fulfill within the same molecule, both catalytic superoxide generating and cytotoxic activities, compounds **B1**, **C6** and **C9** were the most appropriate for further anticancer testing. Compound **B1**, a diselenide which fulfilled both criteria, was evaluated by flow cytometric analysis for its effects on cell cycle distribution and apoptosis induction in MCF-7 cells and its effect in ROS generation in CCRF-CEM cells. The results indicated that **B1** effectively induced cell cycle accumulation at S and G_2_/M phases and a diminution in G_0_/G_1_ phase, accompanied by an increase in SubG_1_ phase cells. Furthermore, **B1** possessed an apoptogenic effect in MCF-7 and CCRF-CEM cell lines. Finally, Se generated oxidative stress is implicated in cell death induced by **B1** in the CCRF-CEM line. In summary, considering all the biological activities compound **B1** should be considered as novel candidates for further studies to understand the exact mechanism of action and for *in vivo* studies.

## Figures and Tables

**Table 1:**
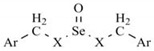
Physical constants for compounds **D1-D8**.

Ref.	Ar	X	Yield (%)	M.p. (°C)	Recryst. Solvent	Molecular formula	CHN (%) Calcd/Found
**D1**	*p*-chlorophenyl	S	9	126–128	Methanol	C_14_H_14_Cl_2_OS_2_Se	C, 40.77/40.54 H, 3.39/3.48 N, 0.00/0.00
**D2**	*p*-methoxyphenyl	S	14	96–98	Methanol	C_16_H_18_O_3_S_2_Se	C, 47.88/48.10 H, 4.49/4.76 N, 0.00/0.00
**D3**	*p*-trifluoromethylphenyl	S	11	131–133	Methanol	C_16_H_12_F_6_OS_2_Se	C, 40.25/40.51 H, 2.52/2.68 N, 0.00/0.00
**D4**	*p*-cyanophenyl	S	18	170–172	Methanol	C_16_H_12_N_2_OS_2_Se	C, 49.10/49.37 H, 3.07/3.35 N, 0.00/0.00
**D5**	*p*-nitrophenyl	S	7	124–126	Methanol	C_14_H_12_N_2_O_5_S_2_Se	C, 38.98/39.10 H, 2.78/2.60 N, 0.00/0.00
**D6**	*p*-chlorophenyl	NH	22	245–247	Ethanol	C_14_H_14_Cl_2_N_2_OSe	C, 44.68/44.49 H, 3.72/3.40 N, 7.44/7.56
**D7**	*p*-methoxyphenyl	NH	39	176–178	Ethanol	C_16_H_20_N_2_O_3_Se	C, 52.32/52.40 H, 5.45/5.40 N, 7.62/7.37
**D8**	*p*-trifluoromethylphenyl	NH	8	159–161	Ethanol	C_16_H_14_F_6_N_2_OSe	C, 43.34/43.49 H, 3.16/3.10 N, 6.32/6.30

**Table 2:**
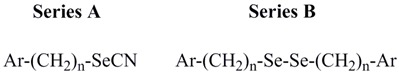
Chemiluminescence (CLU) and cytotoxic activities (IC_50_) of the compounds **A1**-**A13** and **B1**-**B13**.

Compound	Ar	n	CLU^a^	PC-3 cell line	MCF-7 cell line
				IC_50_ (μM)	IC_50_ (μM)
**A1**	4-aminophenyl	0	0.242	10.0	>10
**A2**	4-(*N*,*N*-dimethylamino)phenyl	0	1.095	2.0	>10
**A3**	4-amino-3-carboxyphenyl	0	0.075	NE^b^	>10
**A4**	4-acetamido-3-carboxyphenyl	0	0.301	> 10	>10
**A5**	4-bromophenyl	1	0.372	> 10	>10
**A6**	phenyl	1	0.814	NE	>10
**A7**	4-nitrophenyl	1	0.091	8.9	>10
**A8**	4-trifluoromethylphenyl	1	0.798	> 10	>10
**A9**	4-methylthiophenyl	1	0.810	NE	NE
**A10**	4-methylphenyl	1	1.490	NE	NE
**A11**	4-cyanophenyl	1	0.580	> 10	NE
**A12**	naphthyl	1	0.141	6.0	9.4
**A13**	4-nitrophenyl	2	0.232	NE	NE
**B1**	4-aminophenyl	0	3.928	1.7	4.3
**B2**	4-(*N*,*N*-dimethylamino) phenyl	0	0.281	> 10	>10
**B3**	4-amino-3-carboxyphenyl	0	0.129	NE	>10
**B4**	4-acetamido-3-carboxyphenyl	0	0.085	NE	NE
**B5**	4-bromophenyl	1	0.315	NE	NE
**B6**	phenyl	1	1.543	NE	NE
**B7**	4-nitrophenyl	1	0.141	NE	NE
**B8**	4-trifluoromethylphenyl	1	0.735	> 10	>10
**B9**	4-methylthiophenyl	1	1.089	> 10	>10
**B10**	4-methylphenyl	1	0.570	NE	NE
**B11**	4-cyanophenyl	1	0.119	NE	NE
**B12**	naphthyl	1	0.101	NE	NE
**B13**	4-nitrophenyl	2	0.176	5.0	>10
**Control**			0.103	-	-
**Diselenodipropionic acid**			2.526	-	-
**MSA^c^**			-	8.4 [40]	-
**Etoposide**			-	13.6 ± 2.2 [41]	17.5 ± 1.2 [42]

^a^ CLU are average CLU/30 sec integrals (N = 20); ^b^ No effect; ^c^ Methylseleninic acid.

**Table 3:**
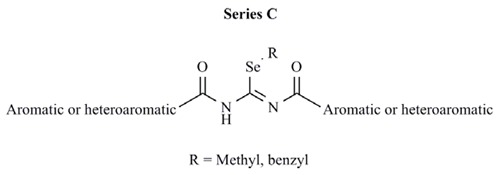
Chemiluminescence (CLU) and cytotoxic activities (IC_50_) of the compounds **C1**-**C25**.

Comp.	Aromatic or heteroaromatic	R	CLU^a^	PC-3 cell line	MCF-7 cell line
				IC_50_ (μM)	IC_50_ (μM)
**C1**	3-pyridyl	methyl	0.110	>10	6.2
**C2**	4-methylphenyl	methyl	0.157	1.8	3.0
**C3**	4-cyanophenyl	methyl	0.085	>10	0.1
**C4**	3,5-dimethoxyphenyl	methyl	0.040	6.5	2.8
**C5**	2-chloro-3-pyridyl	methyl	0.221	9.1	1.3
**C6**	2-furyl	methyl	2353	7.4	>10
**C7**	2-thienyl	methyl	0.165	6.1	>10
**C8**	3-chloro-2-thienyl	methyl	0.517	6.3	8.3
**C9**	5-nitro-3-thienyl	methyl	2760	1.2	6.0
**C10**	2-isoxazolyl	methyl	0.408	7.6	0.6
**C11**	2-benzothienyl	methyl	ND^b^	> 10	>10
**C12**	3,4-methylenedioxybenzyl	methyl	0.043	8.1	>10
**C13**	3-quinolinyl	methyl	0.100	7.6	>10
**C14**	2-phenyl-4-quinolinyl	methyl	0.054	7.7	NE
**C15**	9-acridinyl	methyl	0.185	> 10	NE
**C16**	2-furyl	benzyl	508	> 10	>10
**C17**	2-thienyl	benzyl	0.073	NE^c^	NE
**C18**	3-chloro-2-thienyl	benzyl	0.052	NE	>10
**C19**	5-nitro-3-thienyl	benzyl	0.063	> 10	>10
**C20**	2-isoxazolyl	benzyl	0.216	NE	>10
**C21**	2-benzothienyl	benzyl	0.076	> 10	>10
**C22**	3,4-methylenedioxybenzyl	benzyl	0.046	NE	NE
**C23**	3-quinolinyl	benzyl	0.051	> 10	NE
**C24**	2-phenyl-4-quinolinyl	benzyl	0.061	> 10	>10
**C25**	9-acridinyl	benzyl	0.051	> 10	NE
**Control**			0.060	-	-
**Diselenodipropionic acid**			2.526	-	-
**MSA^d^**			-	8.4 [40]	-
**Etoposide**			-	13.6 ± 2.2 [41]	17.5 ± 1.2 [42]

^a^ CLU are average CLU/30 sec integrals (N = 20); ^b^ Not determined; ^c^ No effect; ^d^ Methylseleninic acid.

**Table 4:**
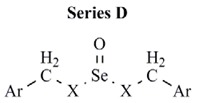
Chemiluminescence (CLU) and cytotoxic activities (IC_50_) of the compounds **D1-D8**.

Comp.	Ar	X	CLU^a^	PC-3 cell line	MCF-7 cell line
				IC_50_ (μM)	IC_50_ (μM)
**D1**	*p*-chlorophenyl	S	0.160	NE^b^	NE
**D2**	*p*-methoxyphenyl	S	890	NE	NE
**D3**	*p*-trifluoromethylphenyl	S	1451	NE	>10
**D4**	*p*-cyanophenyl	S	701	NE	NE
**D5**	*p*-nitrophenyl	S	4743	NE	NE
**D6**	*p*-chlorophenyl	NH	0.072	3.9	8.4
**D7**	*p*-methoxyphenyl	NH	0.562	NE	>10
**D8**	*p*-trifluoromethylphenyl	NH	1973	NE	NE
**Control**			0.137	-	-
**Diselenodipropionic acid**			2.526	-	-
**MSA^c^**			-	8.4 [40]	-
**Etoposide**			-	13.6 ± 2.2 [41]	17.5 ± 1.2 [42]

^a^ CLU are average CLU/30 sec integrals (N = 20); ^b^ No effect; ^c^ Methylseleninic acid.

**Table 5:** Spectroscopic data (IR, ^1^H-NMR and MS) for the new compounds.

Ref.	IR (KBr; υ/cm^−1^)	^1^H NMR (400 MHz, DMSO-*d*_6_, δ/ppm, *J* in Hz)	MS 70 eV; *s*; (% abundance)
**D1**	3030	4.75 (d, 4H, 2 -S-CH_2_-), 7.32 (d, 4H, H_3_ + H_5_ + H_3’_ + H_5’_), 7.46 (d, 4H, H_2_ + H_6_ + H_2’_ + H_6’_).	125 (100)
**D2**	3003	3.73 (s, 6H, 2OCH_3_), 4.84 (s, 4H, 2 -S-CH_2_-), 6.87 (d, 4H, H_3_ + H_5_ + H_3’_ + H_5’_), 7.17 (d, 4H, H_2_ + H_6_ + H_2’_ + H_6’_).	90 (100)
**D3**	3008	5.04 (d, 4H, 2 -S-CH_2_-), 7.62 (d, 4H, H_3_ + H_5_ + H_3’_ + H_5’_ ), 7.73 (d, 4H, H_2_ + H_6_ + H_2’_ + H_6’_).	159 (100)
**D4**	3061	5.03 (d, 4H, 2 -S-CH_2_-), 7.59 (d, 4H, H_3_ + H_5_ + H_3’_ + H_5’_ ), 7.85 (d, 4H, H_2_ + H_6_ + H_2_ + H_6’_).	116 (100)
**D5**	3112	4.98 (d, 4H, 2 -S-CH_2_-), 7.75 (d, 4H, H_3_ + H_5_ + H_3’_ + H_5’_ ), 8.16 (d, 4H, H_2_ + H_6_ + H_2’_ + H_6’_).	136 (100)
**D6**	3324	4.21 (d, 4H, 2 –NH-CH_2_-), 6.54 (t, 2H, 2-NH-CH_2_-), 7.28 (d, 4H, H_3_ + H_5_ + H_3’_ + H_5’_ ), 7.36 (d, 4H, H_2_ + H_6_ + H_2’_ + H_6’_).	140 (100)
**D7**	3351-3321	3.73 (s, 6H, 2OCH_3_), 4.14 (d, 4H, 2 –NH-CH_2_-), 6.28 (t, 2H, 2-NH-CH_2_-), 6.87 (d, 4H, H_3_ + H_5_ + H_3’_ + H_5’_ ), 7.17 (d, 4H, H_2_ + H_6_ + H_2’_ + H_6’_).	136 (100)
**D8**	3335	4.32 (d, 4H, 2 –NH-CH_2_-), 6.69 (t, 2H, 2-NH-CH_2_-), 7.46 (d, 4H, H_3_ + H_5_ + H_3’_ + H_5’_ ), 7.68 (d, 4H, H_2_ + H_6_ + H_2’_ + H_6’_).	174 (100)
